# Inhibitory Effects of *Schisandra* Lignans on Cytochrome P450s and Uridine 5′-Diphospho-Glucuronosyl Transferases in Human Liver Microsomes

**DOI:** 10.3390/pharmaceutics13030371

**Published:** 2021-03-10

**Authors:** Hyung-Ju Seo, Seung-Bae Ji, Sin-Eun Kim, Gyung-Min Lee, So-Young Park, Zhexue Wu, Dae Sik Jang, Kwang-Hyeon Liu

**Affiliations:** 1 BK21 FOUR Community-Based Intelligent Novel Drug Discovery Education Unit, College of Pharmacy and Research Institute of Pharmaceutical Sciences, Kyungpook National University, Daegu 41566, Korea; seohj1992@naver.com (H.-J.S.); wltmdqo2377@naver.com (S.-B.J.); hjkopsty@gmail.com (S.-E.K.); lgm00179@naver.com (G.-M.L.); soyoung561021@gmail.com (S.-Y.P.); 2 Mass Spectrometry Based Convergence Research Institute and Department of Chemistry, Kyungpook National University, Daegu 41566, Korea; wuzhexue527@gmail.com; 3 Department of Life and Nanopharmaceutical Sciences, Kyung Hee University, Seoul 02447, Korea

**Keywords:** *Schisandra chinensis*, lignans, cytochrome P450, uridine 5′-diphosphoglucuronosyl transferase, drug interaction

## Abstract

*Schisandra chinensis* has been widely used as a traditional herbal medicine to treat chronic coughs, fatigue, night sweats, and insomnia. Numerous bioactive components including lignans have been identified in this plant. Lignans with a dibenzocyclooctadiene moiety have been known to possess anti-cancer, anti-inflammatory, and hepatoprotective activity. Fragmentary studies have reported the ability of some lignans to modulate some cytochrome P450 (P450) enzymes. Herein, we investigated the drug interaction potential of six dibenzocyclooctadiene lignans (schisandrin, gomisin A, B, C, and N, and wuweizisu C) on nine P450 enzymes (CYP1A2, 2A6, 2B6, 2C8, 2C9, 2C19, 2D6, 2E1, and 3A) and six uridine 5′-diphosphoglucuronosyl transferase (UGT) enzymes (UGT1A1, 1A3, 1A4, 1A6, 1A9, and 2B7) using human liver microsomes. We found that lignans with one or two methylenedioxyphenyl groups inhibited CYP2B6, CYP2C8, CYP2C9, CYP2C19, and CYP2E1 activities in a time- and concentration-dependent like their CYP3A inhibition. In comparison, these lignans do not induce time-dependent inhibition of CYP1A2, CYP2A6, and CYP2D6. The time-dependent inhibition of gomisin A against CYP2C8, CYP2C19, and CYP3A4 was also elucidated using glutathione as a trapping reagent of reactive carbene metabolites given that gomisin A strongly inhibits these P450 enzymes in a time-dependent manner. A glutathione conjugate of gomisin A was generated in reactions with human recombinant CYP2C8, CYP2C19, and CYP3A4. This suggests that the time-dependent inhibition of gomisin A against CYP2C8, CYP2C9, and CYP3A4 is due to the production of carbene reactive metabolite. Six of the lignans we tested inhibited the activities of six UGT to a limited extent (IC_50_ > 15 μM). This information may aid the prediction of possible drug interactions between *Schisandra* lignans and any co-administered drugs which are mainly metabolized by P450s.

## 1. Introduction

Herb drug interactions (HDI) which result in serious adverse events have received significant attention with the increased use of alternative medicines as well as the widespread use of combination therapies for various diseases in recent years [1,2]. The underlying mechanisms of HDI mainly involve the modulation of cytochrome P450 (P450) and uridine 5′-diphosphoglucuronisyl transferase (UGTs) enzyme activities [2,3]. For example, Saint John’s Wort, a well-studied example of such an HDI, and hyperforin is thought to be the main component that modulates CYP3A and CYP2C9 enzymes [4]. Many well-known herbal medicines including ginseng [5,6], ginkgo [7], green tea [8], and *Schisandra* [9] have also been reported to result in pharmacokinetic drug interactions with clinical drugs. In a recent study, curcuma was found to significantly increase the urine metabolic ratio of dextromethorphan/dextrorphan by inhibiting CYP2D6 [10]. 

**Figure 1:**
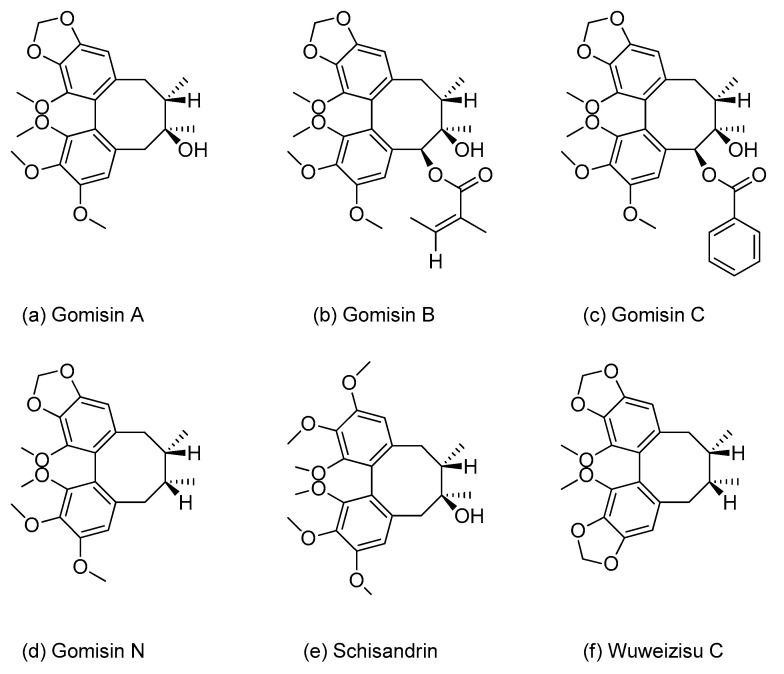
Chemical structures of the six lignans: (**a**) gomisin A, (**b**) gomisin B, (**c**) gomisin C, (**d**) gomisin N, (**e**) schisandrin, and (**f**) wuweizisu C.

*Schisandra chinensis* Bailon (Schisandraceae), a climbing plant distributed in Asia (Korea, Japan, and China) [11] and its fruits, known as omija in Korea, have been extensively used in traditional herbal medicine to treat chronic coughs, enuresis, fatigue, night sweats, and insomnia [12]. In clinical settings, however, co-administration of *Schisandra* extracts or their components with other therapeutic drugs may lead to HDIs. For example, *Schisandra* extracts markedly increased the blood concentration of taclolimus by inhibiting the CYP3A enzyme in liver transplant patients [9]. Gomisin N, one of the most abundant lignans isolated from the fruits of *S. chinensis*, has also been shown to increase the oral bioavailability of drugs metabolized by CYP3A, including midazolam in rats [13]. Therefore, dibenzocyclooctadiene lignans, the major active components of *S. chinensis*, may account for some HDIs. The major lignans in the fruits of *S. chinensis* are gomisin A, -B, -C, and -N, as well as schisandrin and wuweizisu C (Figure 1), with schisandrin being the most abundant, accounting for 33−45% of the total lignans in omija (*S. chinensis*) fruits [14,15,16].

A few in vitro studies investigating the modulatory effects of individual dibenzocyclooctadiene lignans on P450 enzyme activities have been carried out. For example, previous studies found that gomisin A, -C, and -G inhibit CYP3A-mediated midazolam 1′-hydroxylation with IC_50_ values of 1.86 μM, 0.059 μM, and 0.19 μM in a recombinant CYP3A4 isoform, respectively [17,18], while schisandrin A inhibits CYP2C19-catalyzed omeprazole hydroxylation with an IC_50_ of 86.4 μM in recombinant CYP2C19 [19]. Moreover, schisandrin and gomisin A were found to inhibit recombinant CYP3A4 activity with IC_50_ values of 32.0 and 1.39 μM, respectively [20]. Iwata et al. (2004) also evaluated the effects of six lignans including schisandrin, gomisin A, and -N on P450 activities, however, their inhibitory effects were estimated for only five P450s (1A2, 2C9, 2C19, 2D6, and 3A) [21]. Recent studies have discussed the clinical significance of CYP2A6, CY2B6, CYP2C8, and CYP2E1, in addition to the five major P450s, with respect to drug interactions [22]. For example, clopidogrel [23] and piperine [24] were shown to cause a significant increase in the plasma levels of montelukast and chlorzoxazone via inhibition of CYP2C8 and CYP2E1, respectively. Cho et al. (2016) previously suggested that rifampin significantly enhances CYP2B6-mediated efavirenz 8-hydroxylation in humans by inducing CYP2B6 activity [25].

Compared to the studies on P450-mediated drug interaction with lignans, data on the inhibitory effects of lignans on UGT enzymes are rare, even though UGT inhibition is regarded as one of the most important factors for clinical HDIs [26]. The only relevant available data are on the inhibitory potential of schisandrin A and gomisin C against UGT activities [27].

Although some studies have investigated the inhibitory effects of several dibenzocyclooctadiene lignans on P450 and UGT, there are not enough published data to compare the inhibitory effects of individual lignans on P450 and UGT enzyme activities in human liver microsomes (HLMs). Thus, the goals of our study were three-fold: (1) to evaluate the inhibitory potential of six lignans on nine P450s and six UGTs in HLMs; (2) to investigate the time-dependent inhibition of six lignans on nine P450s in HLMs; and (3) to elucidate the time-dependent inhibition mechanism of gomisin A in recombinant P450s (rP450s).

## 2. Materials and Methods 

### 2.1. Chemicals and Reagents 

Acetaminophen, *N*-acetylserotonin (AS), amodiaquine, bupropion, chenodeoxycholic acid (CDCA), chlorzoxazone, *N*-desethylamodiaquine, dextromethorphan, dextrorphan, 6-hydroxybupropion, 7-hydroxycoumarin, estrone-β-D-glucuronide, naloxone, naloxone-β-D-glucuronide, phenacetin, trifluoperazine, trifluoperazine-β-D-glucuronide, trimipramine, alamethicin, uridine diphosphoglucuronic acid (UDPGA), nicotinamide adenine dinucleotide phosphate (NADP^+^), glucose-6-phosphate (G6P), glucose-6-phosphate dehydrogenase (G6PDH), and glutathione (GSH) were purchased from Sigma-Aldrich (St. Louis, MO, USA). *N*-Acetylserotonin-β-D-glucuronide, coumarin, chenodeoxycholic acid-24-acyl-β-glucuronide, dehydronifedipine, diclofenac, 7-ethyl-10-hydroxy camptothecin (SN-38) glucuronide, 4-hydroxydiclofenac, 4-hydroxymephenytoin, 6-hydroxychlorzoxazone, 6β-hydroxytestosterone, midazolam, *S*-mephenytoin, mycophenolic acid (MPA), mycophenolic acid-β-D-glucuronide, nifedipine, and testosterone were purchased from Toronto Research Chemicals (Toronto, ON, Canada). We obtained 1′-hydroxymidazolam and 7-ethyl-10-hydroxy camptothecin (SN-38) from Cayman Chemical (Ann Arbor, MI, USA) and Santa Cruz Biotechnology (Dallas, TX, USA), respectively. Nicotinamide adenine dinucleotide phosphate (NADPH) was provided by Oriental Yeast (Tokyo, Japan). Pooled HLMs (XTreme 200, H2630, mixed gender) were supplied by XenoTech (Lenexa, KS, USA). We purchased rP450 isoforms (rCYP1A2, rCYP2A6, rCYP2B6, rCYP2C8, rCYP2C9, rCYP2C19, rCYP2D6, rCYP2E1, and rCYP3A4) from SPMED (Busan, Korea). All solvents used in the analyses were LC-MS grade (Fisher Scientific Co., Pittsburgh, PA, USA).

We isolated gomisin A, gomisin N, schisandrin, and wuweizisu C from fruits of *Schisandra chinensis* Bailon. The four compounds were purified and analyzed by high-performance liquid chromatography system (HPLC) to obtain 95% pure samples. Chemical structures of the isolated compounds were determined by NMR. All structures were consistent with previously published data [28]. Gomisin B (95%) and gomisin C (98%) were purchased from Toronto Research Chemical (Toronto, ON, Canada) and Sigma-Aldrich, respectively. We adopted the nomenclature of lignans from the recent review article by Opletal et al. (2004) [29].

### 2.2. Inhibitory Effects of Six Lignans against Human Cytochrome P450 Activity 

The inhibitory potential of six lignans on nine P450 activities was evaluated as previously described with slight modifications [30]. Lignans were first dissolved in methanol. The final organic solvent concentration in the incubation media was ≤1.0% (*v/v*). The microsomal incubation was conducted using two cocktail sets containing non-interactive substrates: set A containing phenacetin, bupropion, amodiaquine, diclofenac, *S*-mephenytoin, and dextromethorphan as substrates for CYP1A2, CYP2B6, CYP2C8, CYP2C9, CYP2C19, and CYP2D6, respectively; and set B containing coumarin and chlorzoxazone as substrates for CYP2A6 and CYP2E1, and midazolam, nifedipine, and testosterone as substrates for CYP3A4 (Table 1). These substrates are known to be selective for each P450 isoforms. Incubation mixtures containing potassium phosphate buffer (0.1 M, pH 7.4), pooled HLMs (0.25 mg/mL), P450 probe substrate cocktails, and lignan (0~50 μM) were pre-incubated at 37 °C (5 min). Following pre-incubation, an NADPH generating system (1.3 mM NADP^+^, 3.3 mM G6P, 1 unit/mL G6PDH, and 3.3 mM MgCl_2_) was added to initiate the reaction, and further incubated for 10 min. The reaction was quenched with 50 μL of cold acetonitrile containing internal standard (IS; 7 nM trimipramine). After centrifugation at 14,000 rpm (5 min at 4 °C), aliquots of supernatants were filtered through a 0.2 μm membrane filter and were measured by liquid chromatography-tandem mass spectrometry (LC-MS/MS). All microsomal incubations were performed in triplicate.

For IC_50_ shift assay [31], each lignan (0~50 μM) was pre-incubated with HLMs in the presence of an NADPH generating system at 37 °C for 30 min. After pre-incubation, P450 probe substrate cocktails were added to start the reaction, and further incubated at 37 °C for 10 min. Incubation was terminated by the addition of 50 μL ice-cold acetonitrile containing IS. After centrifugation, aliquots of supernatants were measured by LC-MS/MS. 

### 2.3. Inhibitory Effects of Gomisin A against Recombianat CYP2C8, CYP2C19, and CYP3A4 Activity

The incubation mixture consisted of 0.1 M potassium phosphate buffer (pH 7.4), 20 pmol/mL rCYP2C8, rCYP2C19, or rCYP3A4 enzyme, respective probe substrate and gomisin A (0~50 μM) in a final volume of 100 μL. After a 5 min pre-incubation period at 37 °C, reactions were initiated by the addition of an NADPH generating system, and further incubated for 10 min at 37 °C. For time-dependent inhibition studies, gomisin A (0~50 μM) was pre-incubated with rP450s in the presence of an NADPH generating system at 37 °C for 30 min. After pre-incubation, P450 probe substrates (0.1 μM amodiaquine for CYP2C8, 40 μM *S*-mephenytoin for CYP2C19 or 0.1 μM midazolam for CYP3A4) were added to start the reaction, and further incubated at 37 °C for 10 min. Other conditions remained as described above.

### 2.4. Inhibitory Effects of Six Lignans against Human Uridine-5-Diphosphoglucuronosyl Transferase Activity

The inhibitory potential of six lignans on six UGT activities was evaluated using the previously developed cocktail method with slight modifications [32]. The microsomal incubation was conducted using two cocktail sets containing non-interactive substrates: set A with SN-38, CDCA, and trifluoperazine as substrates for UGT1A1, UGT1A3, and UGT1A4, respectively; and set B containing AS, MPA, and naloxone as substrates for UGT1A6, UGT1A9, and UGT2B7 (Table 2). These substrates are known to be selective for each UGT isoforms. The incubation mixtures containing Tris buffer (0.1 M, pH 7.4), pooled HLMs (0.25 mg/mL), alamethicin (25 μg/mL), UGT probe substrate cocktails, and lignan (0~50 μM) were pre-incubated at 37 °C. After pre-incubation, 5 mM UDPGA was added to initiate the reaction, and further incubated for 60 min. The reaction was quenched with 50 μL of cold acetonitrile containing IS (350 nM estrone-β-D-glucuronide). After centrifugation, aliquots of supernatants were measured by LC-MS/MS.

### 2.5. Characterization of Reactive Metabolites of Gomisin A in Recombinant P450 Isoforms

Gomisin A (50 μM) was incubated for 120 min at 37 °C with rP450s (20 pmol/mL) in 0.1 M phosphate buffer (pH 7.4) in the presence of 2 mM NADPH and 5 mM GSH. Control incubations in the absence of NADPH and GSH were conducted. Incubation samples were quenched with a half-fold volume of cold methanol. After centrifugation, supernatants were concentrated under vacuum and reconstituted with methanol (100 μL). Samples were analyzed by ultra-performance liquid chromatography-high resolution mass spectrometry (UPLC-HRMS).

### 2.6. LC-MS/MS Analysis

The IS and each P450- and UGT-isoform-specific metabolites were separated on a Kinetex XB-C18 column (100 × 2.1 mm, 2.6 μm, Phenomenex, Torrance, CA, USA) and analyzed using a Shimadzu LC-MS 8060 triple-quadrupole mass spectrometer (Shimadzu, Kyoto, Japan) equipped with a Nexera X2 ultra HPLC system (Shimadzu) coupled with an electrospray ionization (ESI) interface. The mobile phase was composed of water containing 0.1% formic acid (A) and acetonitrile containing 0.1% formic acid (B). Elution conditions for the analysis of metabolites of P450 probe substrates were set as 8% B for 0–0.5 min, 8%→60% B for 0.5–5 min, 60% B for 5–6 min, 60%→8% B for 6–6.1 min, and 8% B for 6.1–9 min [30], while that of UGT probe substrates was set as 0% B for 0–1 min, 50% B for 1–5 min, and 0% B for 5.1–8 min [32]. The flow rate was set to 0.2 mL/min. ESI was performed in negative-ion mode at −3500 V or in positive ion mode at 4000 V. For quantitation of each metabolite, the analysis was performed in selected reaction monitoring (SRM) with the precursor-to-product ion transition (Table 1 and Table 2).

To determine the GSH adducts produced by rP450s, a Vanquish UPLC system coupled with a QExactive Focus Orbitrap mass spectrometer (Thermo Fisher Scientific Inc., Waltham, MA, USA) was used. A Kinetex C18 column (100 × 2.1 mm, 2.6 μm) was used to separate the samples. The mobile phase was composed of water containing 0.1% formic acid (A) and acetonitrile containing 0.1% formic acid (B). The gradient elution was set as 10% B for 0–3 min, 10%→98% B for 3–10 min, 98%→10% B for 10–10.1 min, 10% B for 10.1–12 min. The flow rate was set to 0.2 mL/min. Data acquisition was carried out at *m/z* 100–800 with a resolution of 70,000 in the total ion scan mode, and MS/MS spectra were acquired at *m/z* 50–750 at a resolution of 17,500 in the daughter ion scan mode [33]. Parallel reaction monitoring (PRM) was also employed, and the PRM transition *m/z* 710.2576 was used for the detection of GSH conjugate [34]. Instrument settings were as follows: normalized collision energy (CE), 25 eV; capillary temperature, 320 °C; spray voltage, 3.5 kV; sheath gas flow rate, 40 arb; auxiliary gas flow rate, 10 arb; S-lens RF level, 50.0 V. Nitrogen was used for spray stabilization and as the collision gas in the C-trap.

### 2.7. Data Analysis

All results were acquired from three replicates in different microsomal incubations. Analytical data were processed by Shimadzu LabSolutions LC-MS software (Shimadzu, Kyoto, Japan) or Thermo Xcalibur software (Thermo Fisher Scientific Inc.). IC_50_ values were determined by nonlinear regression analysis using WinNonlin software (Pharsight, Mountain View, CA, USA). 

## 3. Results and Discussion

### 3.1. Inhibition of Cytochrome P450 Activities by Six Lignans

The inhibitory potential of the six Omija lignans (gomisin A, -B, -C, and -N, as well as schisandrin, and wuweizisu C) against P450 enzyme activity was evaluated in HLMs (Table 3). Lignans with one methylenedioxyphenyl group such as gomisin A, -B, -C, and -N most strongly inhibited CYP3A activity with IC_50_ of 1.8–2.3, 0.28–0.42, 0.19–0.30, and 1.3–4.5 μM, respectively. The inhibitory potential (IC_50_ = 2.3 μM) of gomisin A on CYP3A-mediated testosterone hydroxylation was similar to previously published data (*K_i_* = 1.01 μM) [35]. The inhibitory potential (IC_50_ = 4.5 μM) of gomisin N on CYP3A-mediated midazolam hydroxylation was also similar to the previously reported value (IC_50_ = 5.5 μM) [36]. The inhibition of eight other P450 isoforms was much lower (IC_50_ > 10 μM) than on CYP3A (IC_50_ ≤ 4.5 μM). Iwata et al. (2004) also reported weak inhibition (IC_50_ > 10 μM) of gomisin A, -B, -C, and -N on CYP1A2, CYP2C9, CYP2C19, and CYP2D6 [21]. Schisandrin without a methylenedioxyphenyl group showed moderate inhibition (IC_50_ ≤ 16 μM) on CYP1A2, CYP2B6, CYP2C19, CYP2E1, and CYP3A, while it showed weak inhibition (IC_50_ = 22.0 ~ 43.0 μM) on other P450s. We found that IC_50_ schisandrin inhibits CYP3A with IC_50_ values of 10.5–16.0 μM, slightly lower than the the 32.0 μM reported by Wan et al. (2010) [20]. This discrepancy could be due to differences in incubation conditions perhaps in the CYP3A probe substrates (midazolam and nifedipine versus fluorescent substrate) and/or the enzyme source (HLM vs rCYP3A4). Wuweizisu C with two methylenedioxyphenyl groups moderately inhibited CYP2B6, CYP2C9, CYP2C19, and CYP3A, while it weakly inhibited CYP1A2, CYP2A6, CYP2C8, CYP2D6, and CYP2E1 (IC_50_ > 20 μM).

All lignans tested weakly inhibited CYP2D6-mediated dextromethorphan *O*-demethylation and CYP2A6-mediated coumarin hydroxylation with IC_50_ values of over 20 μM and 38 μM, respectively. Lignans without a methylenedioxyphenyl group induced stronger inhibition of CYP1A2 and CYP2E1 activities (IC_50_ ≅ 4 μM) than lignans with a methylenedioxyphenyl group (IC_50_ > 25 μM). Gomisin B and gomisin C with a bulky angeloyl or benzoyl group at position 6 showed the strongest inhibitory effect on CYP3A-mediated testosterone hydroxylase activity (IC_50_ = 0.19 ~ 0.28 μM) similar to Iwata et al.’s findings (IC_50_ = 0.26 ~ 0.62 μM) [21], In comparison, gomisin A and gomisin N lacking this functional group showed moderate inhibition (IC_50_ = 1.3 ~ 4.5 μM). Similar inhibitory effects of these four gomisin compounds (gomisin A, -B, -C, and -N) on CYP3A were also demonstrated when assessed by determining midazolam hydroxylation and nifedipine oxidation activities as an index activity of CYP3A (IC_50_ = 0.26 ~ 0.42 μM). Iwata et al. (2004) also reported substrate independent inhibitory effects of these gomisin compounds on CYP3A activity [21]. Gomisin B and gomisin C with a bulky group at position 6 more strongly inhibited CYP2C8-mediated amodiaquine *O*-demethylase activity with IC_50_ values of 10.9 μM and 16.5 μM, respectively, compared to gomisin A and gomisin N lacking this functional group at this position (IC_50_ > 29 μM). Wuweizisu C containing two methylenedioxyphenyl groups induced the strongest inhibition of CYP2B6, CYP2C9, and CYP2C19 (IC_50_ = 2.7 ~ 8.9 μM) compared to the five other lignans. This suggests that the additional methylenedioxyphenyl group influences inhibition of these three P450s.

In addition, several P450 inhibitors including clopidogrel [37], furafylline [38], and ticlopidine [37] have been shown to be time-dependent inhibitors of P450. We investigated the effect of incubation time on the IC_50_ values of six lignans on nine P450s (Table 3). A test compound with an IC_50_ fold-shift decrease ≥ 1.5 is considered to be a time-dependent inhibitor as recommended by Awortwe et al. [39]. Previous studies have shown that gomisin A, -B, -C, and -N inhibit CYP3A activity in a time- and NADPH-dependent manner when co-incubated with HLMs or rP450s [21,35,36]. Our data confirm and elaborate on previous findings allowing us to characterize the time-dependent inhibition of CYP2B6, CYP2C8, CYP2C9, CYP2C19, and CYP2E1 by dibenzocyclooctadiene-based lignans for the first time. Similar to published data [21,35,36], we found that lignans with a methylenedioxyphenyl group inhibit CYP3A and CYP2E1 in a time-dependent manner with IC_50_ shift values > 2.0. Gomisin A, gomisin N, and wuweizisu C without a bulky angeloyl or benzoyl group showed time-dependent inhibition of CYP2C9-mediated diclofenac hydroxylase activity and CYP2C19-mediated mephenytoin hydroxylase activity with IC_50_ shift values > 1.8, whereas gomisin B and gomisin C containing a bulky angeloyl or benzoyl functional group showed time-independent inhibition. Unlike the other five lignans, wuweizisu C containing two methylenedioxyphenyl groups also displayed time-dependent inhibition of CYP2B6-catalyzed bupropion hydroxylase activity with an IC_50_ shift value of 2.1. Inactivation of CYP2C8 is only time-dependent when exposed to lignans with one methylenedioxyphenyl group. The inhibitory potential of schisandrin without a methylenedioxyphenyl group against nine P450s in HLMs pre-incubated in the presence of NADPH did not increase compared to the untreated HLMs. This suggests that the presence of the methylenedioxyphenyl group is a prerequisite for dibenzocyclooctadiene-based lignans to display time-dependent inhibition of P450s. Several studies have also reported the time-dependent inhibition of P450s by compounds containing dibenzocyclooctadiene group (i.e., paroxetine, noscapine, and gomisin C) [21,40,41]. None of the lignans tested showed time-dependent inhibition of CYP1A2, CYP2A6, or CYP2D6 enzymes.

### 3.2. Inhibitory Effects of Gomisin A against Recombinant CYP2C8, CYP2C19, and CYP3A4

Gomisin A displayed the strongest time-dependent inhibition of CYP2C8, CYP2C19, and CYP3A with IC_50_ values ≤ 5.0 μM. We further investigated the inhibitory effects of gomisin A against these three P450s using rP450s. Much like HLMs, gomisin A inhibited CYP2C8, CYP2C19, and CYP3A with IC_50_ values of 30.4 μM, 11.3 μM, and 1.51 μM, respectively, in the absence of an NADPH generating system (Table 3 and Table 4). The inhibitory potential of gomisin A on CYP3A-mediated midazolam hydroxylation was similar to the previously reported value (IC_50_ = 1.86 μM) [18]. Gomisin A showed time-dependent inhibition of CYP2C8-mediated amodiaquine demethylase activity, CYP2C19-mediated *S*-mephenytoin hydroxylase activity, and CYP3A-mediated midazolam hydroxylase activity with an IC_50_ shift of 9.2, 2.3, and 3.0, respectively. IC_50_ value shifts in rP450s were also similar to those in HLMs (Table 3 and Table 4). 

### 3.3. Inhibition of UGT Enzyme Activities by Six Lignans

The inhibitory potential of the six lignans against six UGTs was evaluated using HLMs (Table 5). Gomisin C inhibited UGT1A1 and UGT1A3 activities with IC_50_ values of 24.0 µM and 15.0 µM, respectively, while it had negligible inhibition (IC_50_ > 50 μM) on the other UGTs. The inhibitory potential of gomisin C for UGT1A3 was similar to previously published data (IC_50_ = 12.5 μM) [27]. Gomisin B also inhibited UGT1A1 and UGT1A3 activities with IC_50_ values of 20.7 µM and 16.5 µM, respectively.

### 3.4. Characterization of Reactive Metabolites of Gomisin A in Recombinant P450 Isoforms

Methylenedioxyphenyl compounds are converted to reactive intermediates known as carbene metabolites by P450-mediated metabolism. These carbene metabolites easily react with P450 to form a metabolite-intermediate complex (MIC). The formation of this MIC has been reported to play an important role in the time-dependent inhibition of P450 by methylenedioxyphenyl compounds [21,40,42,43]. Dibenzocyclooctadiene-based lignans also generate MIC because they contain a methylenedioxyphenyl group. Iwata et al. previously showed that gomisin C inactivates CYP3A4 by forming an MIC with CYP3A4 [21]. Gomisin N and P450-induced carbene reactive metabolite formation has also been demonstrated [33]. GSH can be used as trapping agent to identify carbene reactive metabolites because carbene is unstable and cannot be detected directly [44,45,46]. In this study, gomisin A showed time-dependent inhibition of CYP2C8-mediated amodiaquine demethylase activity with an IC_50_ shift of 10.5 and 9.2 in HLMs and rCYP2C8, respectively–the highest observed values in this study. In addition, we found that gomisin A displayed strong time-dependent inhibition of CYP2C8, CYP2C19, and CYP3A4 in HLMs and rP450s (IC_50_ < 5 µM).

**Figure 2:**
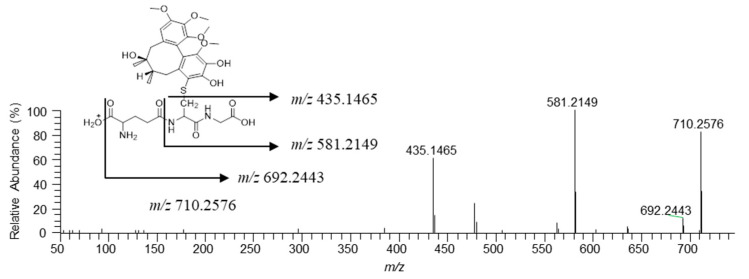
MS/MS spectrum of glutathione conjugate of gomisin A annotated with the proposed structures of fragment ions obtained by UPLC-HRMS analysis of the human recombinant CYP2C8 incubates of gomisin A in the presence of NADPH generating system and glutathion.

**Figure 3:**
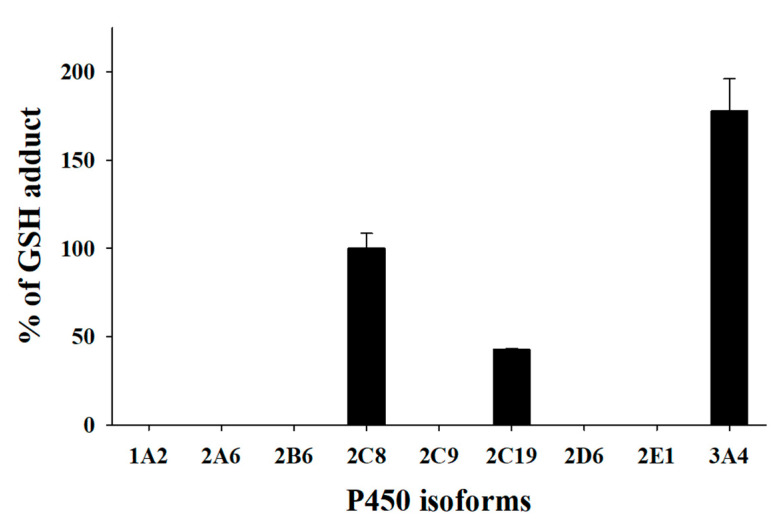
Representative plots for the formation of the glutathione adduct of gomisin A by human recombinant cytochrome P450 enzymes (rP450s). The incubation system (100 μL) contained 0.1 M phosphate buffer solution (pH 7.4), 1 pmol P450 isoforms, 50 μM gomisin A, 2 mM NADPH, and 5 mM glutathione. Each data represent the mean ± SD of triplicate determinations.

To elucidate the time-dependent inhibition mechanism of gomisin A against CYP2C8, CYP2C19, and CYP3A4, gomisin A was incubated with rP450s in the presence of NADPH and GSH. UPLC-HRMS analyses indicated that there was only one GSH conjugate ([M+H]^+^, *m/z* 710.2576, t_R_ = 6.2 min) formed in rCYP2C8. UPLC-HRMS analyses of the peak responsible for this GSH conjugate displayed a protonated molecule [M+H]^+^ at *m/z* 710.2576 (mass error < 2 ppm), 293 Da higher than that of gomisin A. This suggests that gomisin A first loses [-CH_2_] before it is conjugated with one molecule of GSH [47]. The MS/MS spectrum of the GSH conjugate by fragmenting *m/z* 710.2576 through collision gave the characteristic daughter ions at *m/z* 692.2443, 581.2149, and 435.1485, suggesting the loss of a water molecule (−18 Da), the loss of a pyroglutamate residue (−129 Da) of GSH, and cleavage of the cysteinyl C-S bond (−275 Da) (Figure 2). The fragment ions observed from the loss of a pyroglutamate residue and cleavage of the cysteinyl C−S bond are the most typical ions found in GSH conjugates [33,44,48,49]. A further experiment was carried out to identify the P450 isoforms involved in the bioactivation of gomisin A using the PRM method. The results showed that CYP2C8, CYP2C19, and CYP3A4 were involved in the formation of carbene reactive metabolite of gomisin A (Figure 3).

### 3.5. Evaluation of Drug Interaction Potential of Six Lignans

In previous studies, *Schisandra* extracts and lignans were found to alter the pharmacokinetics of drugs which are substrates of CYP3A. Animal studies show that oral *Wuzhi* capsules (including 0.14 mg/g schisandrin, 0.09 mg/g gomisin A, 5.79 mg/g gomisin C, 0.63 mg/g schisanhenol, and 5.69 mg/g deoxyshisandrin) significantly increase blood tacrolimus concentration through CYP3A inhibition [50]. Wang et al. (2014) reported that in rats, *Schisandra chinensis* alcoholic extracts (containing 1.84% schisandrin, 1.54% gomisin A, 2.43% deoxyshisandrin, and 1.23% gomisin N) with tacrolimus exert a greater increase on tacrolimus’s *C*_max_ and AUC values than when used alone, thereby indicating inhibition of CYP3A, a major tacrolimus-metabolizing enzyme [51]. Deoxyshisandrin, a CYP3A inhibitor, markedly increases plasma concentrations of midazolam in rats [52]. The magnitude of AUC variation for drugs that are predominantly biotransformed by CYP3A4 was estimated to increase by 22–321% in the presence of gomisin C, a strong CYP3A inhibitor [17].

In contrast to the extensive studies on drug interactions with CYP3A substrates, there is a paucity of data detailing drug interactions with other P450s. We predicted the clinical herb drug interaction risk induced by *Schisandra* lignans based on each of the inhibitory potential. Gomisin A inhibited CYP2C8 activity with an IC_50_ value of 2.8 μM, similar to CYP3A inhibition (IC_50_ = 0.77~1.2 μM) in a time- and concentration-dependent manner. Considering that gomisin A participates in the pharmacokinetic intervention of cyclophosphamide by blocking CYP3A-mediated metabolism and reducing chloroacetaldehyde production in rats [35], gomisin A may interact with CYP2C8 substrate drugs such as amodiaquine [53], paclitaxel [54], and repaglinide [55]. In rats, oral administration of gomisin N for 3 days also resulted in a significant increase in midazolam AUC values [13]. Gomisin N might also interact with CYP2C19 substrate drugs, such as clopidogrel [56] and omeprazole [57], because its CYP2C19 inhibitory potential (IC_50_ = 3.5 μM) is similar to CYP3A inhibition (IC_50_ = 1.7 μM). In vivo studies are required to determine the clinical relevance of potential herb drug interactions between typical doses of *Schisandra* extracts, including lignans, and CYP2C8 or CYP2C19 substrate drugs.

## 4. Conclusions

In conclusion, we report that lignans containing one or two methylenedioxyphenyl groups inhibit CYP2B6, CYP2C8, CYP2C9, CYP2C19, and/or CYP2E1 in a time- and concentration-dependent manner which is similar to CYP3A inhibition. We found that six lignans inhibit six UGTs to a limited extent (IC_50_ > 15 μM). Moreover, our data show that gomisin A inhibits CYP2C8, CYP2C19, and CYP3A4 enzymes in a time-dependent manner by forming carbene reactive metabolites in a similar fashion to gomisin C-induced inhibition of CYP3A4. Additionally, these lignans may result in clinically relevant pharmacokinetic interactions with other co-administered drugs biotransformed by CYP2B6, CYP2C8, CYP2C9, CYP2C19, and/or CYP2E1.

## Figures and Tables

**Table 1:** Optimized selected reaction monitoring (SRM) parameters for metabolites of the nine P450 substrates and internal standard (IS).

P450 Enzyme	Substrate	Concentration (μM)	Metabolite	SRM Transition (*m/z*)	Polarity *	Collision Energy (eV)
1A2	Phenacetin	20	Acetaminophen	152 > 110	ESI^+^	25
2A6	Coumarin	1	7-Hydroxycoumarin	163 > 107	ESI^+^	17
2B6	Bupropion	3	6-Hydroxybupropion	256 > 238	ESI^+^	10
2C8	Amodiaquine	0.1	*N*-Desethylamodiaquine	328 > 283	ESI^+^	13
2C9	Diclofenac	1	4-Hydroxydiclofenac	312 > 231	ESI^+^	15
2C19	*S*-Mephenytoin	40	4-Hydroxymethenytoin	235 > 150	ESI^+^	15
2D6	Dextromethorphan	2	Dextrorphan	258 > 157	ESI^+^	30
2E1	Chlorzoxazone	5	6-Hydroxychlorzoxazone	184 > 120	ESI^−^	18
3A4	Midazolam	0.1	1′-Hydroxymidazolam	342 > 203	ESI^+^	28
	Nifedipine	0.2	Dehydronifedipine	345 > 284	ESI^+^	30
	Testosterone	2	6β-Hydroxytestosterone	305 > 269	ESI^+^	15
IS	Trimipramine	0.007	-	295 > 100	ESI^+^	17

* ESI: Electrospray ionization (ESI) interface to generate protonated molecular ion [M+H]^+^ or deprotonated molecular ion [M-H]^−^.

**Table 2:** Optimized selected reaction monitoring (SRM) parameters for the metabolites of the six UGT substrates and internal standard (IS).

UGT Enzyme	Substrate	Concentration (μM)	Metabolite	SRM Transition (*m/z*)	Polarity	Collision Energy (eV)
1A1	SN-38 *	0.5	SN-38 glucuronide	569 > 393	ESI^+^	30
1A3	Chenodeoxycholic acid	2	CDCA-24 glucuronide	567 > 391	ESI^-^	20
1A4	Trifluoperazine	0.5	TFP *N*-glucuronide	584 > 408	ESI^+^	30
1A6	*N*-Acetylserotonin	1	*N*-SER glucuronide	395 > 219	ESI^+^	10
1A9	Mycophenolic acid	0.2	MPA 7-*O*-glucuronide	495 > 319	ESI^-^	25
2B7	Naloxone	0.2	NX 3-glucuronide	504 > 310	ESI^+^	30
IS	Estrone-β-D-glucuronide	0.25		445 > 269	ESI^-^	35

* SN-38: 7-Ethyl-10-hydroxy camptothecin; SI: Electrospray ionization (ESI) interface to generate protonated molecular ion [M+H]^+^ or deprotonated molecular ion [M-H]^−^.

**Table 3:** Inhibitory effects of six lignans against nine cytochrome P450 enzymes. All incubations were performed in triplicate (*n* = 3).

P450 Enzyme	Probe Substrate	IC_50_ (µM)																	
		Gomisin A			Gomisin B			Gomisin C			Gomisin N			Schisandrin			Wuweizisu C		
		RI *	TDI **	IC_50_ Shift	RI	TDI	IC_50_ Shift	RI	TDI	IC_50_ Shift	RI	TDI	IC_50_ Shiaft	RI	TDI	IC_50_ Shift	RI	TDI	IC_50_ Shift
1A2	Phenacetin	37.4	>50	-	>50	>50	-	>50	>50	-	>50	>50	-	4.0	>50	-	25.6	22.4	<1.5
2A6	Coumarin	>50	>50	-	>50	>50	-	>50	>50	-	38.2	>50	-	40.9	>50	-	>50	>50	-
2B6	Bupropion	>50	>50	-	42.6	>50	-	33.1	>50	-	38.3	>50	-	14.7	>50	-	2.9	1.4	2.1
2C8	Amodiaquine	29.3	2.8	10.5	16.5	4.9	3.4	10.9	5.8	1.9	31.7	9.9	3.2	22.0	>50	-	21.0	14.6	<1.5
2C9	Diclofenac	45.4	22.0	2.1	45.7	>50	-	>50	>50	-	36.2	10.9	3.3	43.0	>50	-	8.9	3.6	2.5
2C19	*S*-Mephenytoin	11.2	4.8	2.3	>50	37.8	-	16.3	20.4	-	10.4	3.5	3.0	5.3	46.1	-	2.7	1.5	1.8
2D6	Dextromethorphan	45.7	>50	-	45.5	>50	-	>50	>50	-	42.0	>50	-	40.2	>50	-	20.3	28.2	-
2E1	Chlorzoxazone	>50	15.6	>3.2	>50	20.6	>2.4	>50	24.4	>2.0	>50	23.6	>2.1	4.2	36.0	-	>50	25.2	>2.0
3A	Midazolam	3.1	1.2	2.6	0.42	0.12	3.5	0.30	0.10	3.0	4.5	1.7	2.7	10.5	35.0	-	25.9	2.5	10.4
	Nifedipine	1.8	0.77	2.3	0.32	0.10	3.2	0.26	0.09	2.9	1.4	0.61	2.4	16.0	43.3	-	5.6	1.2	4.7
	Testosterone	2.3	0.77	3.0	0.28	0.09	3.1	0.19	0.09	2.1	1.3	0.55	2.4	5.8	20.6	-	3.6	1.2	3.0

* RI: Reversible inhibition, ** TDI: Time-dependent inhibition.

**Table 4:** Inhibitory effects of gomisin A against CYP2C8, CYP2C9, and CYP3A4 isoforms in human recombinant P450 isoforms.

Recombinant P450 Enzyme	Probe Substrate	IC_50_ (μM) *		
		Gomisin A		
		RI **	TDI **	IC_50_ shift
rCYP2C8	Amodiaquine	30.4 ± 8.1	3.32 ± 1.05	9.2
rCYP2C19	*S*-Mephenytoin	11.3 ± 3.3	4.98 ± 0.49	2.3
rCYP3A4	Midazolam	1.51 ± 0.20	0.51 ± 0.07	3.0

* Values represent the average ± S.E. of triplicate. ** RI: Reversible inhibition, TDI: Time-dependent inhibition.

**Table 5:** Inhibitory effects of six lignans against six uridine 5′-diphosphoglucuronosyl transferase (UGT) enzymes.

UGT Enzyme	Substrate	IC_50_ (μM) *					
		Gomisin A	Gomisin B	Gomisin C	Gomisin N	Schisandrin	Wuweizisu C
1A1	SN-38 **	>50	20.7	24.0	>50	>50	>50
1A3	Chenodeoxycholic acid	>50	16.5	15.0	26.9	>50	>50
1A4	Trifluoperazine	>50	>50	>50	>50	>50	>50
1A6	*N*-Acetylserotonin	>50	>50	>50	>50	>50	>50
1A9	Mycophenolic acid	>50	>50	>50	>50	>50	>50
2B6	Naloxone	>50	>50	>50	>50	>50	>50

* Values represent the average in triplicate; ** SN-38: 7-Ethyl-10-hydroxy camptothecin.

## Data Availability

All data in this study have been included in this manuscript.
